# Development of a Cost-Effective Optical Sensor for Continuous Monitoring of Turbidity and Suspended Particulate Matter in Marine Environment

**DOI:** 10.3390/s19204439

**Published:** 2019-10-14

**Authors:** T. Matos, C. L. Faria, M. S. Martins, Renato Henriques, P. A. Gomes, L. M. Goncalves

**Affiliations:** 1 MEMS-UMinho, University of Minho, Campus de Azurém, 4800-471 Guimarães, Portugal; carlosfaria@dei.uminho.pt (C.L.F.); mmartins@dei.uminho.pt (M.S.M.); 2 LARSyS, University of Algarve Campus de Gambelas, 8005-139 Faro, Portugal; 3 Institute of Earth Sciences, University of Minho, Campus de Gualtar, 4710-057 Braga, Portugal; rhenriques@dct.uminho.pt; 4 Centre of Molecular and Environmental Biology (CBMA), University of Minho, 4710-057 Braga, Portugal; pagomes@bio.uminho.pt

**Keywords:** in-situ measurement, oceanography, suspended particulate matter, turbidity optical sensor

## Abstract

A cost-effective optical sensor for continuous in-situ monitoring of turbidity and suspended particulate matter concentration (SPM), with a production cost in raw materials less than 20 €, is presented for marine or fluvial applications. The sensor uses an infrared LED and three photodetectors with three different positions related to the light source—135º, 90º and 0º—resulting in three different types of light detection: backscattering, nephelometry and transmitted light, respectively. This design allows monitoring in any type of environment, offering a wide dynamic range and accuracy for low and high turbidity or SPM values. An ultraviolet emitter–receiver pair is also used to differentiate organic and inorganic matter through the differences in absorption at different wavelengths. The optical transducers are built in a watertight structure with a radial configuration where a printed circuit board with the electronic signal coupling is assembled. An in-lab calibration of the sensor was made to establish a relation between suspended particulate matter (SPM) or the turbidity (NTU) to the photodetectors’ electrical output value in Volts. Two different sizes of seashore sand were used (180 µm and 350 µm) to evaluate the particle size susceptibility. The sensor was tested in a fluvial environment to evaluate SPM change during sediment transport caused by rain, and a real test of 22 days continuous in-situ monitoring was realized to evaluate its performance in a tidal area. The monitoring results were analysed, showing the SPM change during tidal cycles as well as the influence of the external light and biofouling problems.

## 1. Introduction

In both oceanography and limnology, turbidity is of great importance in the study of the development conditions of the euphotic zone, where the passage of sunlight is crucial for the development of marine flora [1], study of the sediments transport phenomena [2,3] and study of water quality [4], particularly those for human consumption. Turbidity is a physical property of fluids that translates into reduced optical transparency due to the presence of suspended and dissolved materials that block the passage of light [5]. These materials can be of organic or inorganic origin, varying in colour, matter and size, ranging from macroscopic to colloidal particles [6]. 

**Figure 1:**
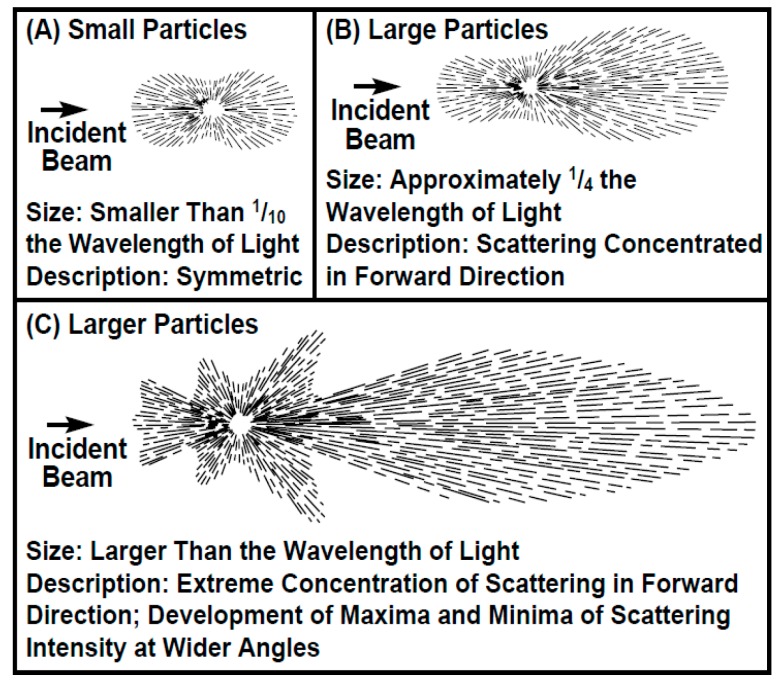
Light scattering principle. In (**A**), when the particle size is 1/10 of the wavelength of the incident light, the diffusion is practically symmetrical. For particles four times smaller than the wavelength, the diffusion has a greater intensity in the direction in which the light propagates (**B**). In (**C**), for particles larger than the wavelength of incident light, the intensity becomes even greater in the direction of light propagation [8].

Turbidity is not a physical quantity that is directly measurable. The current optical turbidity sensors make use of the light absorption and scattering from suspended sediments to perform a correlation with the turbidity value. Particulate materials primarily attenuate light through scattering but can also have a significant contribution to total absorption. Dissolved substances scatter negligibly but do attenuate light through absorption. Considering this, a device to measure suspended particulate matter concentration (SPM) or turbidity should be based at least on both optical properties. Light absorption is a process by which light is absorbed and converted into energy by the optical block material and is related to the decrease of luminous energy in its directional path. Scattering is the physical process where the light is forced to deviate from its straight trajectory by one or more paths due to particles or other non-uniformities in the medium (see Figure 1). The interactions between the optical properties of suspended materials such as colour and size; the characteristics of the fluid, especially its index of refraction, colour and properties of the solutes; and the wavelength and intensity of the incident light, make the turbidity a visual property that is quite complex [7].

The first practical attempt to measure turbidity in-lab was through the Jackson candle method. Developed over a century ago, this instrument consists of a lighted candle placed under a glass tube with a flat bottom. The fluid in which the turbidity is to be measured is slowly poured into the tube until the flame image is no longer visible from the point of view of the top (the light does not disappear completely, just the image of the flame).

Other turbidimeters based on the extinction of light were developed. Among these, the most recognized and still used today in naval instruction is the Secchi disk, created in 1865 by Pietro Angelo Secchi [9]. The Secchi disk consists of a flat circular disk with a diameter between 16 cm and 40 cm, which is usually divided into four equivalent parts with the contrasts of black and white or, in some cases, is completely black or completely white [10]. This disc, attached by a rope, is slowly submerged in the water until it is no longer visible, finding the Secchi depth (usually in meters or centimetres). High depths are related to high clarity of water and low levels of turbidity. Oppositely, low depths indicate high levels of turbidity. The readings of this instrument depend on the attenuation of the light in the water, that is, the ability of the light to penetrate the medium. Even though it is a widely used method, it has many uncertainties that need to be considered during analysis. Readings are affected by changes in sunlight conditions, water shaking, time of day and human error [11]. Due to these factors, electronic instruments were developed, offering greater dynamic range and accuracy.

The optical turbidimeters have solved the problem of susceptibility to human error presented by previous methods. This type of device uses a light source and one or more optical receivers. When the light passes through the medium it will be scattered and absorbed by the existing suspended particles, varying the electrical signal of the receivers. The electrical value is then related to the turbidity.

There are several standard water quality standard methods in use. The US Environmental Protection Agency (EPA) has approved eight standards for monitoring drinking water turbidity. Until 2009, only four methods were accepted: EPA Method 180.1 [12], Standard Method 2130B, Great Lakes Instrument Method 2 (GLI 2) [13] and Hach Method [14]. In 2009, USEPA approved four new methods: Mitchell Methods M527 and M5331, Orion AQ4500 and AMI Turbiwell. In addition to these, the United States Geological Survey also uses other methods, such as the International Organization for Standardization (ISO) 7027 [15]. All those methods are used in water for human consumption, offering a high accuracy for low turbidity values. For marine or fluvial environments, where turbidity can have higher values, many of these methods and related devices become impracticable, not only because of their low dynamic range, but also because most of them are laboratory devices, making them large, expensive and dependent of the electrical grid. Some turbidity sensors for continuous monitoring were developed for continuous monitoring in the last years [16,17,18,19]. Most of them are optical devices that only use one type of detection, becoming specific for a strict type of environment and without the flexibility to adapt to other contexts.

As detailed, there are some standardized methods for measuring turbidity, however, each measurement method uses a different unit. A multiplicity of turbidity units has been introduced because a change in the design, type of light source, detector or measuring angle will change the sensor reading. Thus, different turbidity instruments can produce different measurements in the same sample. Boss et al. recommended to stop the use of turbidity standards for assessment of SPM and that efforts should be focused on calibrating with SPM, a biogeochemical variable of direct link to water quality, and if possible, the use of several concurrent optical methods to estimate SPM [20].

Another technology that has gathered attention is the acoustic backscattering (ABS) [21,22,23]. ABS-based devices do not pretend to measure turbidity but SPM and sediments particle sizes. Although it is a promising technology, it still presents low accuracy and, comparing to the optical devices [24], are more expensive and need higher electrical power. On the other hand, optical technologies are deeply affected by biofouling and acoustic ones more easily overcome this problem [25].

Attending to the actual development and state of art of turbidity devices, arises the necessity to develop cost-effective sensors that are low power, robust, with a flexible dynamic range and accuracy to adapt to different environment conditions, and with the necessary conditions to be massively spread in a region, with large spatial resolution and providing in-situ continuous monitoring. The same necessity was reported before by NeXOS project and by the European Marine Board Expert Working Group on Advancing Citizen Science for Coastal and Ocean Research [26]. We present a cost-effective device, based on multi optical methods, and calibrated for both assessment of turbidity (NTU) and SPM measurement.

## 2. Sensor Design

The patented [27] developed sensor aims to measure the turbidity and concentration of suspended particles in marine or fluvial environments.

Illuminating an aqueous sample with undissolved matter, the emitted light is subjected to attenuation, diffraction and reflection caused by the particles that obstruct the passage of light [28]. Using optical transducers (light-emitting source as actuator and photodetectors as receivers), a correlation is established between the electric value sensed by the optical receivers and the turbidity in the sample or its concentration of suspended sediments.

### 2.1. Optical Transducers

The selection of the transducers is a focal point for the correct function of the sensor. The infrared (IR) wavelength was selected (940 nm) due to its lower susceptibility to the colouration of the particles present in the medium. This wavelength is also outside the typical optical absorption range of organic matter (typically ultraviolet, green and blue). Moreover, due to higher water light absorbance in this wavelength when compared to visible wavelength, less ambient light interference is expected when the sensor is submerged.

**Figure 2:**
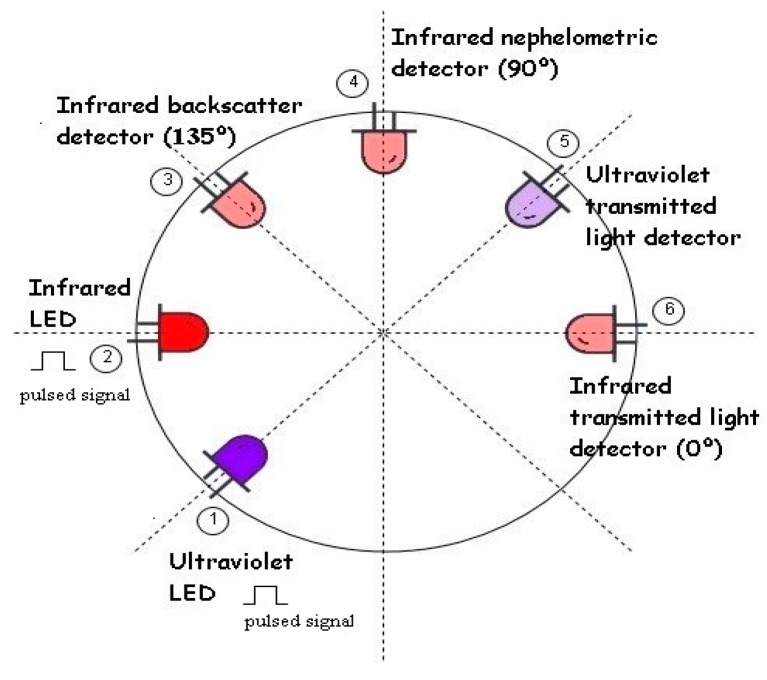
Schematic of the transducers’ positions design. Different receptor positions relative to the light source provide different electrical responses. In the image are represented the IR LED (2) and the three types of detection: backscatter (3), nephelometric (4) and transmitted light (6). The UV emitter (1) and wideband receiver (5) are also presented.

LEDs are used in the light-emitting sources, as shown in Figure 2(2), due to its low cost and wide commercial offering. In addition, compared to the competitors, they have a much faster response than lamps, allowing light to be pulsed at high frequencies. Also, compared to lasers, LEDs require less maintenance and present fewer calibration problems. As optical receivers, to implement the optical-electrical transduction, three phototransistors in different positions related to the emitting source, 135º, 90º and 0º, are used, providing respectively the backscattering, nephelometric and transmitted light detections. 

For the backscattering light detection technique, a photodetector, shown in Figure 2(3), is placed at 135º to the light source, shown in Figure 2 (2), to sense the reflected light back from the sediments suspended in the fluid. For pure water, this type of detection has a zero-optical sensing value (there are no obstacles to reflect the light). With increasing turbidity and a consequent increase of suspended sediments and reflections, the detected light output will also increase. The advantage of this type of detection is the wide measuring range and accuracy for high turbidity values. On the other hand, for low turbidity values, backscattering is not as accurate as nephelometric detection. The backscatter detection strongly depends on the size, composition and shape of the suspended particles [29].

The nephelometric detection measures the diffracted light at 90º, as shown in Figure 2(2),(4). As for the backscatter technique, for pure water, the absence of optical obstacles results in a null optical value, which will increase with the increase of suspended particles. However, for high turbidity values, the reflected light will also be absorbed by the materials and the light output will decrease. The nephelometric detection is particularly accurate for low turbidity but not so useful for high values and it depends mostly on the size and number of particles in suspension [30].

The transmitted light detection is the measurement related to the absorbance of the light and uses an optical detector at 0º to the light source, as shown in Figure 2(2),(6). For distilled water, the detector has a maximum output value that will decrease with the increase of turbidity (particles will absorb and scatter the light on its path). This technique presents higher sensibility, offering a wide dynamic range. On the other hand, it is very vulnerable to the colouration and particle size which results in lower precision [31].

Finally, an ultraviolet (UV) emitter and wideband receiver, as shown in Figure 2(1),(5), are used to distinguish organic from inorganic matter. The suspended load can be from both organic and inorganic origin [32], and drastic changes in organic load can translate into several environmental problems [33]. These two types of matter present different behaviours to different wavelengths: Compared to inorganic matter, organic compounds have higher ultraviolet/infrared absorption ratio [34]. Considering this, using the absorption values of infrared and ultraviolet transmitted light detectors, discrimination between different types of matter may be possible. Instead of UV, other wavelengths could be used, however, since most marine phytoplankton and chlorophyll flourish in green, blue and yellow, it could produce associated errors in the measurements [35].

### 2.2. Hardware

**Figure 3:**
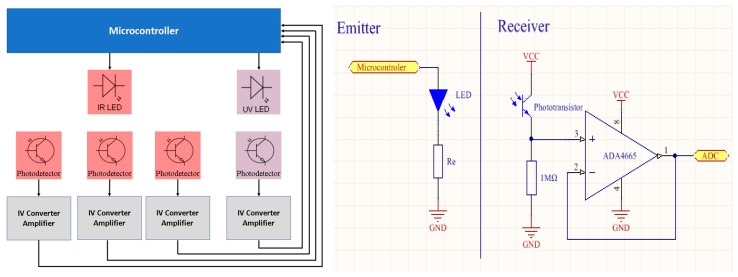
Hardware schematic of the sensor. A printed circuit board with the electronic instrumentation is integrated with the mechanical sensor structure and housing. The emitting hardware uses LEDs controlled by the microcontroller, with a serial resistor Re (different values from IR and UV LEDs to match their own current values) to settle the light intensity. The receiving hardware is composed of the phototransistors and I-V conversion resistors (Rr), and a buffer amplifier to reduce leakage currents when connected to the ADC of the microcontroller.

A small size (2.5 cm × 1.5 cm) printed circuit board with the electronic instrumentation (Figure 3) was designed to be assembled inside the sensor housing, close to the optical receivers. Each photodetector has a current to voltage converter (1MΩ resistor) and a buffer amplifier that allows the reduction of leakage currents and capacitances. The sensor comprises an ultra-low-power microcontroller that controls the IR and UV LEDs and reads the four outputs: backscatter, nephelometric and IR- and UV-transmitted light detectors. Each LED is pulsed ON during 500 µsec. Detector outputs are read during the LEDs on and off periods and the difference is calculated in the microcontroller to minimize ambient light influence. The sensor can be connected to an external device by a simple network cable.

The developed sensor uses an OFL-5102 infrared LED source (940nm) with a radiant intensity of 15 mW at 20 mA and 10º emitting angle. For the ultraviolet source (385nm), the VAOL-5GUV8T4 LED was used, with a luminous intensity of 80 mcd at 20 mA and 30º emitting angle. To match the IR light source, three W53P3C phototransistors were used, with a central wavelength of 940nm, view angle of 20º, and dark current of 100 nA. To match the ultraviolet light source, one TEPT5700 phototransistor was used with a wavelength peak of 540nm (for better selectivity a UV optical filter can also be used), angle sensitivity of 50º and dark current of 3nA. To reduce the leakage capacitance and currents, a rail-to-rail buffer amplifier using the op-amp ADA4665 was implemented, with a reduced bias current (1pA maximum), noise density of 32 nV/√Hz and slew rate of 1 V/us. An STM32L496 microcontroller was used, due to its low power in sleep/standby mode, and an accurate 12-bit channel ADC (Analog to Digital Converter). With this configuration, the turbidity sensor presents a current consumption below 300 µA in sleep mode and 20 mA during readings, with autonomy of 1 year with a 1/min sample rate, using a common mobile-phone 3000 mA x 3.7 V lithium battery. The developed sensor has a production cost bellow 20 €, including hardware and watertight structure materials.

### 2.3. Sensor Housing

**Figure 4:**
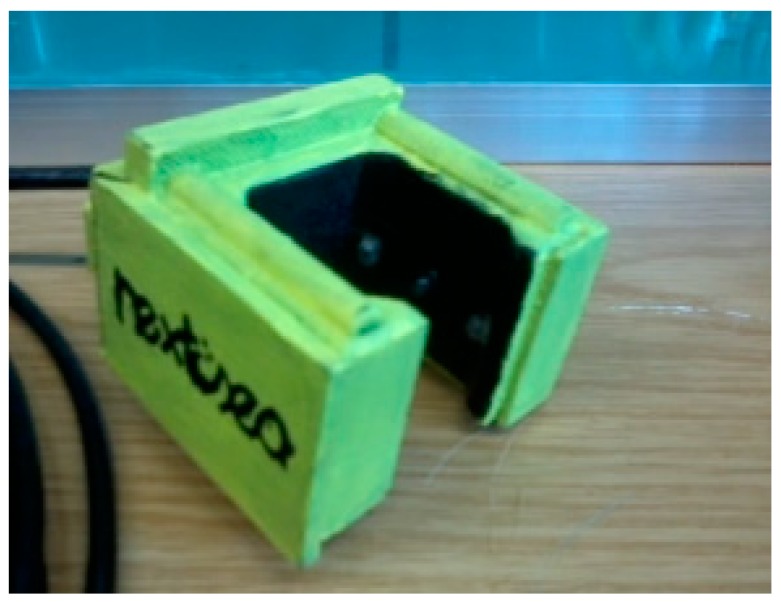
Turbidity optical sensor built in a radial configuration by 3D printing and filled with epoxy for submersion.

The sensor housing (Figure 4) was built by 3D printing, with a radial configuration, to place the transducers in the positions presented in Figure 2. Since the sensor is intended for a long time and massive deployments, some effort was made to avoid harmful effects on marine ecosystems. The structural material is polylactic acid (PLA), a long-time biodegradable maize-based compound. The requirement to be watertight was met since the sensor interior was filled with epoxy resin, protecting the electronics from water infiltration. As an example, Bisphenol An epoxy diacrylate is inherently biodegradable, has no potential for bioaccumulation and is not toxic to aquatic life. The sensor walls, where the LEDs are placed, have high rugosity and are painted with opaque black painting to minimize undesired light reflections.

## 3. In-lab Calibration

In-lab calibrations were conducted to prepare the sensor for in-situ measurements. The mathematical correlation between turbidity or SPM and the electrical value of each photodetector of the sensor is presented, as well the possibility to distinguish between organic and inorganic matter. A calibration methodology to eliminate the external light influence that may affect measurements is also demonstrated. 

To enable the comparison between the developed instrument and the available commercial devices, a 4000 NTU Formazin Turbidity Standard was used to calibrate the sensor to the most popular turbidity unit in use.

The electronics are not expected to be sensitive to temperature, pressure, and long-term drift in LED power or phototransistor sensitivity, however, this was not assessed to date. Also, a test with different salt concentrations (from 0 to 60 g/L and steps of 5g/L) was conducted and no significant changes were detected in sensors output voltage due to the wide field of view and relatively short light path.

### 3.1. SPM Calibration (Inorganic Matter)

As recommend by Gibbs [36], Downing [37], Zaneveld et al. [38] and Boss et al. [20] among others, the calibration was conducted to match the electrical value of the photodetectors to the concentration of suspended sediments (g/L). The concentration of suspended sediments unit was chosen, but an equivalent procedure could be used to calibrate the output to other types of units. Seashore sand was used since monitoring of seashore coastal areas is expected in future deployments by the authors. A calibration for two different sizes of sand (180µm and 350µm) was made to evaluate differences in the responses of the detectors. For better results, calibration must be performed with sediments expected in deployment location. 

A 3 L volume of distilled water was used as the first sample and measurements were made at each increment of 30 g (10 g/L) of sand, up to a maximum of 420 g. For each concentration, 20 measurements were recorded to calculate the mean and standard deviation (see Appendix A). 

**Figure 5:**
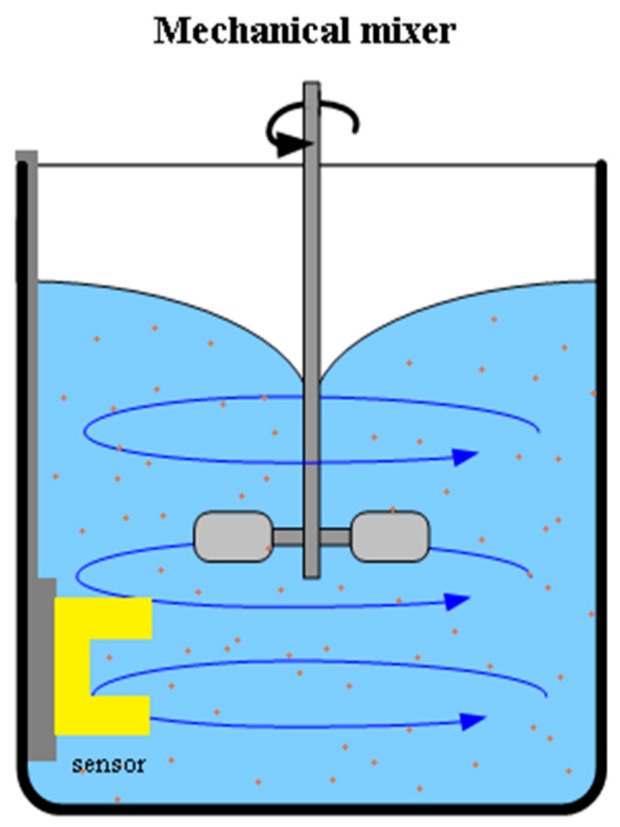
In-lab calibration setup. The measurements were taken in an opaque container to eliminate the external light, and a mixer was used to keep the particles in suspension.

The test setup included an opaque container to eliminate the external light effects and a mechanical mixer to keep the particles suspended and generate a homogeneous mixture (Figure 5).

**Figure 6:**
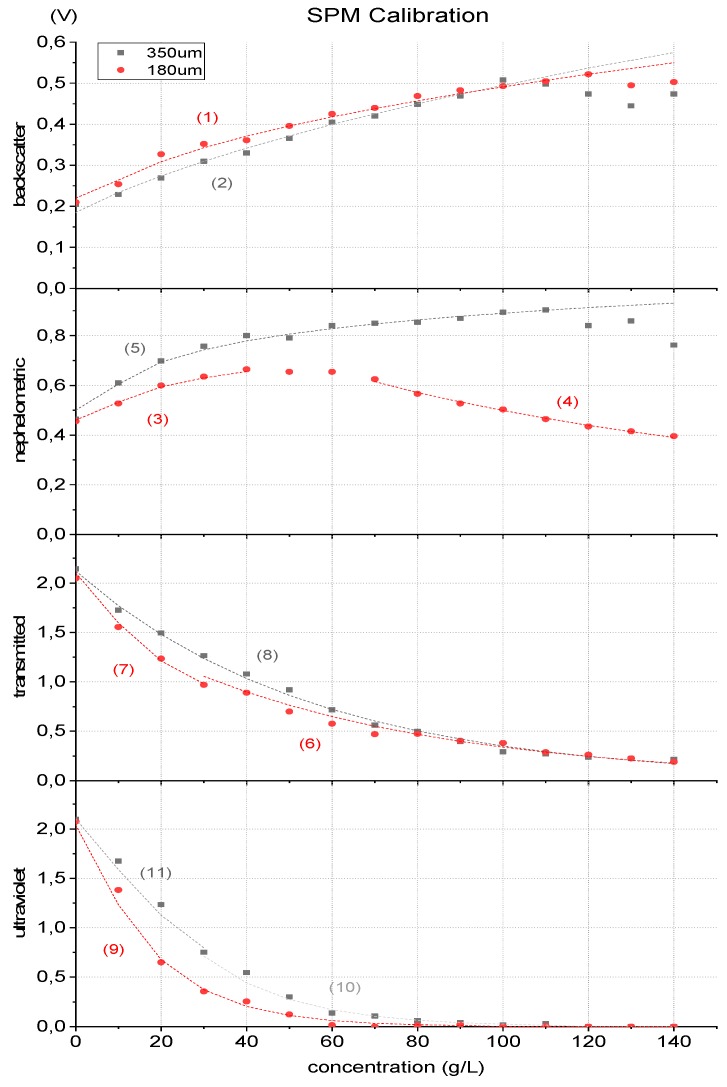
Sensor output voltages for different concentrations of 180 and 350 µm seashore sand. The three IR techniques are presented (backscatter, nephelometric and transmitted light) as the UV transmitted light detection. Mathematical fitting is also presented.

The calibration results for both particle sizes are shown in Figure 6.

The output of the different detectors had the expected behaviour, as described. Some variation in the values is noticed due to the movement of the particles and to the fact that the sample is not totally homogeneous (notice in Appendix A that for the initial water sample the standard deviation is lower than with sediments), however, the concentration-voltage outputs are quite sharp. Analysing each photodetector curve, it is plausible to state that different particle sizes produce different responses, which leads to conclude that the in-situ characteristics should be considered. For each detector, a mathematical expression based on calibration results was developed to correlate the SPM expressed in g/L (pm), as a function of the electrical value output of the receptors in Volt (v), for 180 µm and 350 µm sand, resulting in the following equations:

Backscatter

(1) $$pm\;=\;971.45v^{2}-337.26v+31.269\;,\;\mathrm{to}\;180\;µm$$

(2) $$\;pm\;=\;447.22v^{2}+18.507v-18.772\;\;,\;\mathrm{to}\;350\;µm$$

Nephelometric

(3) $$pm\;=\;0.0258e^{11.194v},\;\mathrm{to}\;180\;µm\;\wedge \;pm<40$$

(4) $$\;pm\;=\;462.49e^{-3.069v},\;\mathrm{to}\;180\;µm\;\wedge \;pm>70$$

(5) $$\;pm\;=\;0.0675e^{8.198v},\;\mathrm{to}\;350\;µm$$

Transmitted IR

(6) pm = −61.66ln(v)+33.313, to 180 µm ∧ v<1.553

(7)  pm = −36.27ln(v)+26.966, to 180 µm ∧ v>1.553

(8) pm = −55.71ln(v)+41.805 , to 350 µm

Transmitted UV

(9) pm = −16.7ln(v)+13.459, to 180 µm ∧ v>0.356

(10) pm = −20.94ln(v)+22.901, to 350 µm ∧ v<0.752

(11) pm = −29.01ln(v)+23.414, to 350 µm ∧ v>0.752

The equations above are used in the next section to process the in-situ data.

### 3.2. Organic Matter

An effective method to quantify organic matter must consider the biological characteristics of the local where the in-situ tests are performed. A generic calibration becomes impractical due to the great variability of absorption properties of the organic matter that may exist [39]. Therefore, a concept proof has been realized to prove that distinguishing organic and inorganic matter may be possible. The sensor was submerged in 400 mL of distilled water, and a solution of phytoplankton was used to increase the organic matter in the sample.

**Figure 7:**
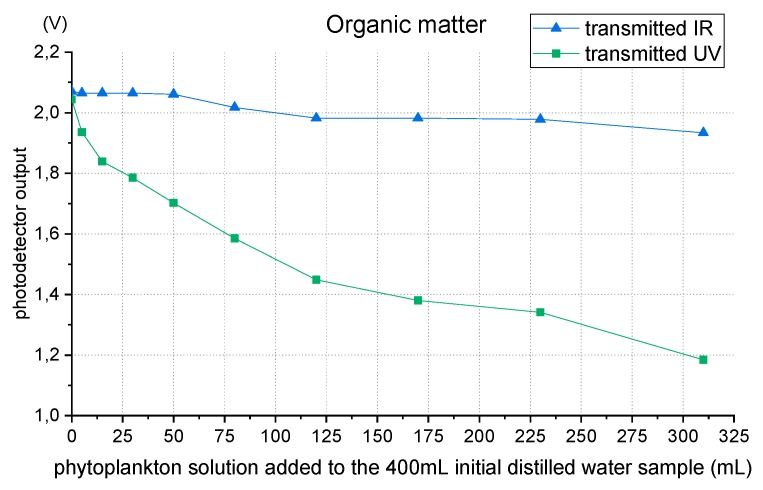
Transmitted IR and UV outputs when organic matter (phytoplankton) is added to water.

The test results are presented in Figure 7.

**Figure 8:**
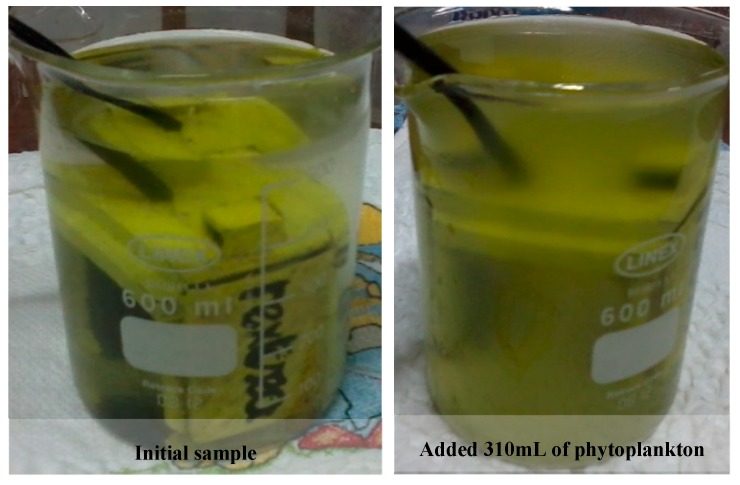
Images of organic matter tests. On the left, the initial solution with 400 mL of distilled water, and on the right, the final sample with 310 mL addition of phytoplankton solution.

Figure 7 shows that, for organic matter, light absorption in the UV range is higher than IR light absorption, as expected. The ultraviolet transmitted light detector presents a higher voltage drop comparing to the infrared one that shows a slow decrease due to the reducing of sample transparency (see Figure 8). By comparing graphs of Figure 6 and Figure 7 results, it is visible that for the same attenuation in IR light transmission, UV light attenuation is higher with organic matter than with non-organic matter.

With the absorption different ratios of ultraviolet and infrared radiation by organic matter, it is possible to perform a quantification of the different types of matter, provided that an effective calibration is made with the local characteristics.

### 3.3. Turbidity NTU Calibration

A 4000 NTU Turbidity Formazin Standard was used to calibrate the sensor to Nephelometric Turbidity Units (NTU). The different NTU samples were performed by diluting the initial 4000 NTU sample in deionized water and following the methods and procedures of the Hach Water Analysis Guide [40]:

(12) $$Dilution_{factor}=\frac{volume_{total}}{volume_{\;sample}}\;=\;\;\frac{volume_{\;deionized\_water}+volume_{\;NTU\_sample}}{volume_{\;NTU\_sample}}$$

**Figure 9:**
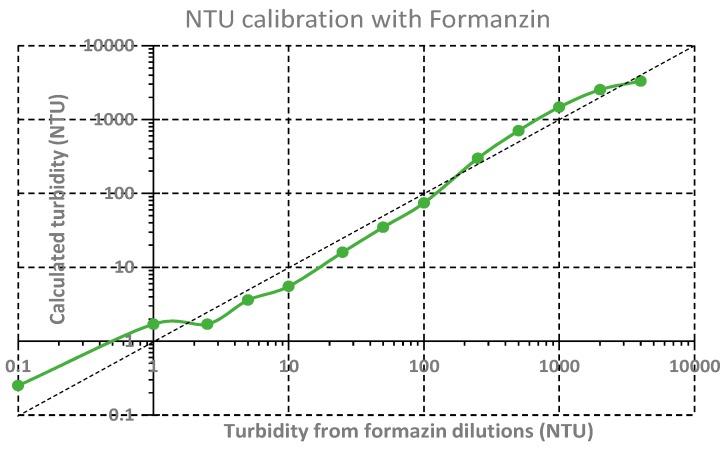
NTU calibration from 0,01 to 4000 NTU formazin turbidity samples taking advantage of the combination of the three different light detections.

Figure 9 shows the NTU calibration results for the four types of light detection combined.

Unlike the SPM calibration, where each detection was treated individually, for the turbidity results, an algorithm was designed to combine the four photodetector outputs (nephelometric, backscatter and both IR and UV transmitted). It is important to notice that both data processing could be used for each calibration (and field tests), as well as other ones like a simple weight filter.

The turbidity output values (“calculated turbidity” in Figure 9) were calculated using the mean value of the three detecting technologies. Each sensor was first scaled to a 0–100% reading. The reading from transmission IR and UV detectors was inverted (0% was calculated with a 100% voltage reading and vice-versa. This was justified since the voltage of backscatter and nephelometric detectors increase with turbidity increases, while for the transmitted technique, voltage decreases when turbidity decreases). The mean of the three detecting technologies was then applied. Turbidity was calculated with a simple linear regression in the logarithmic scale, using the logarithm of sensor output to calculate the logarithm of turbidity (NTU). The dashed line in Figure 9 presents the expected ideal calibration.

The calibration results show that the maximum voltage reading value of the sensor has not been reached with the 4000 NTU Formazin original sample (only the nephelometric detector has achieved its maximum voltage value). In the opposite manner, for the low-level reading of the sensor, the turbidity sensor presents a detectability of 0.1 NTU.

Some commercial turbidity sensors, such as the ECO FLNTU Series from Sea-Bird Scientific [41], claim a 0.01 NTU precision but are limited to 25 or 100 NTU range, only having a good performance in clear water. For example, the Seapoint Turbidity Meter [42], form SeaPoint Sensors, claims a range of 4000 NTU (non-linear above 750 NTU) and a sensitivity of 1 mV/NTU and costs more than 2000 €. The developed sensor reaches the same dynamic range with a cost less than 20 €, with the necessary cost-effectiveness properties for a wide scale replication.

### 3.4. External Light Calibration

During the SPM tests, it was observed that the external light has a clear influence on measurement accuracy. Despite using pulsed light and the difference between output readings during LEDs off and on period, if a light source external to the sensor reaches the photodetector, a voltage offset is produced in photodetectors output. This is due to the non-linearity of current versus light output of the detectors. This effect is very important in in-situ measurements, where the daylight or other natural or unnatural light sources can interfere in the real-sensed turbidity or SPM values.

**Figure 10:**
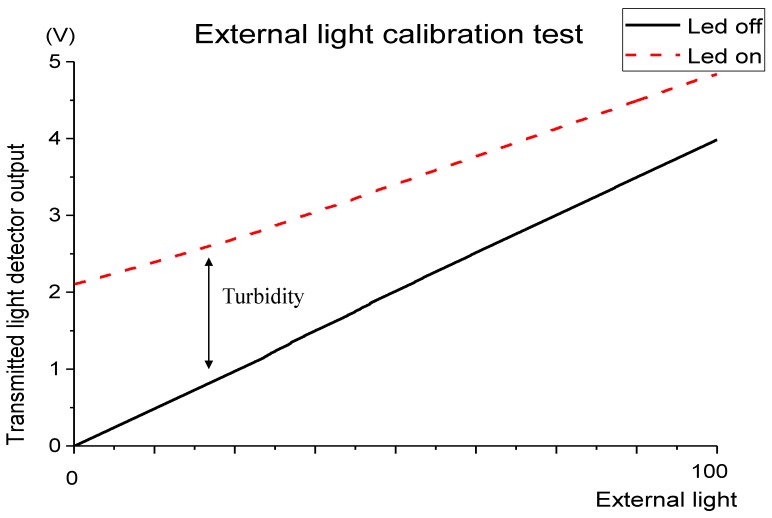
Test results for external light calibration. A sample with constant turbidity was illuminated by an external light source. The detector output voltage with the IR LEDs on (turbidity measurement) and off (external light influence) was registered.

To calibrate the sensor to the external light, it was submerged in water with an external light source gradually illuminating the sample (the offset produced by the external light was increased while the SPM remained the same). Figure 10 presents the infrared transmitted light detector output, as a function of external light (measured with an auxiliary wide-band photodetector), when the infrared LED from turbidity measurement is ON and OFF.

In Figure 10, it is observed that the difference between the turbidity value (LED on) and the offset produced by the external light source (LED off) is not constant due to the light attenuation behaviour. In this way, the sensibility of the turbidity sensor decreases with the increase of the external light. For each photodetector, a mathematical expression was calculated to eliminate the external light effect, with y corresponding to the photodetector correction factor and x the voltage measured by external light influence (measurement with IR LEDs off). On–Off voltage measured is divided by this factor.

Backscatter

(13) $$y\;=\;-0.0049x^{3}+0.0436x^{2}-0.1981x+0.9798$$

Nephelometric

(14) $$y\;=\;-0.0077x^{3}+0.0758x^{2}-0.3299x+0.9868$$

Transmitted IR

(15) $$y\;=\;-0.005x^{4}+0.034x^{3}-0.061x^{2}-0.146x+0.986$$

Transmitted UV

(16) $$y\;=\;-0.012x^{4}+0.127x^{3}-0.436x^{2}+0.349x+0.947$$

Using the mathematical equations above, the turbidity and SPM and turbidity measurements can be corrected. The calibration equations developed are used to process the in-situ data to eliminate the offset caused by any kind of external light (the factor is applied to the electrical on–off value that afterward can be used to estimate the turbidity).

## 4. SPM Test in Fluvial Environment

**Figure 11:**
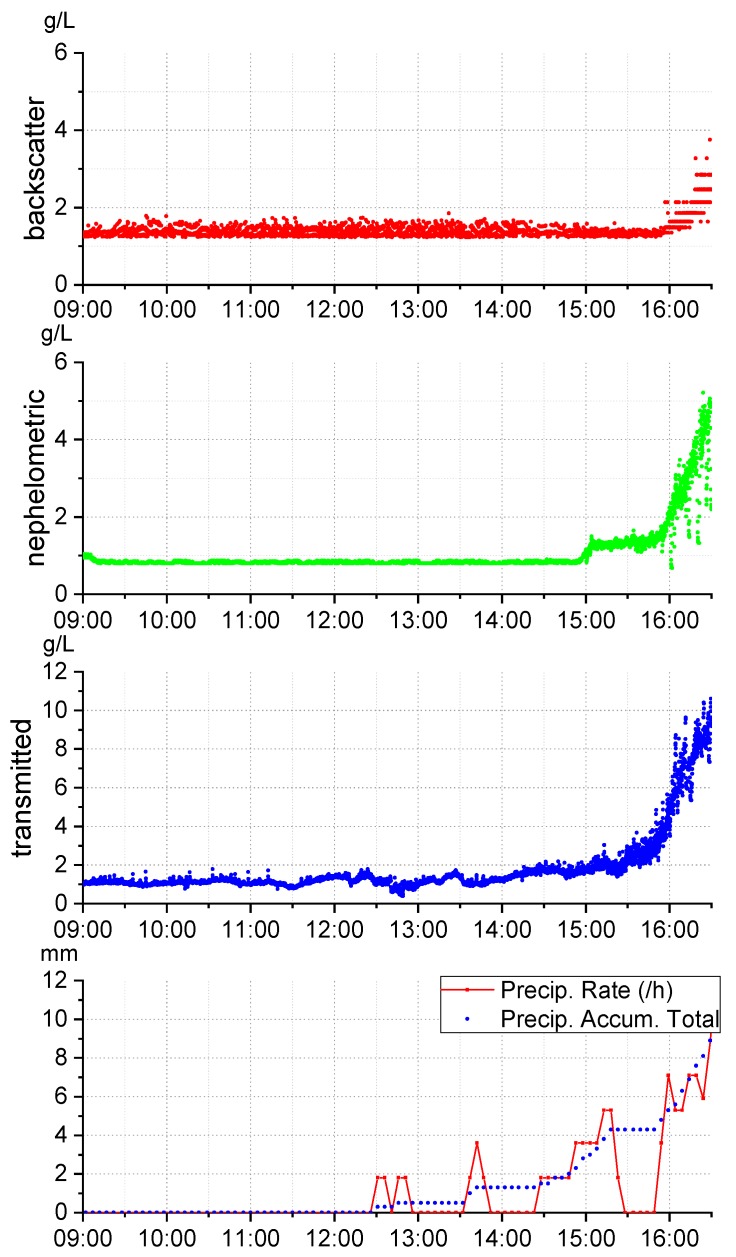
Results of Este river test. In the top graphics, the SPM readings for backscatter, nephelometric and transmitted IR detectors are presented, and in the bottom graphic, the total precipitation accumulation and the precipitation rate during the test are presented.

Tests in a river were conducted to verify SPM variations. The device was installed in Este river (Braga, Portugal, 41°31′37.9″N 8°26′07.3″W) on 27 February 2018, a day when it was expected to rain. The place of the test was strategically chosen on the way out of the city and in a muddy area, where an increase of sediment load was expected during rain, originated by the surrounding mud and city dirt. Previous 180 µm sand calibration and external light compensation was applied. Figure 11 shows the results of SPM change with rain.

The sensor registered an SPM increase with the rain, as expected. The transmitted sensor revealed a higher sensibility, detecting up to 10g/L, comparing to 5g/L and 4g/L of nephelometric and backscatter, respectively. Results may be affected since the river is polluted and, thus, there are several particles with the potential to affect the sense of each phototransistor (remember that colour, size and material have different influences on different light detections). Also, the sensor was calibrated with 180 µm seashore sand, which does not correspond to expected sediments in the river. No variation was detected before rain, from 9AM to 12AM, confirming light dependence compensation technique.

Concluding, the main objective of the test was achieved, registering the river turbidity increase with the rain.

## 5. Marine In-situ Continuous Monitoring

The sensor was deployed for 22 days in-situ continuous monitoring to validate its operation. The test was conducted in Lima river mouth (Viana do Castelo, Portugal, 41°41′49.2″N 8°49′04.8″NW), from 11 July to 1 August 2017, and, for each photodetector, the voltage with LEDs off and on was measured, evaluating the external light and the SPM reading.

### 5.1. External Light Influence

**Figure 12:**
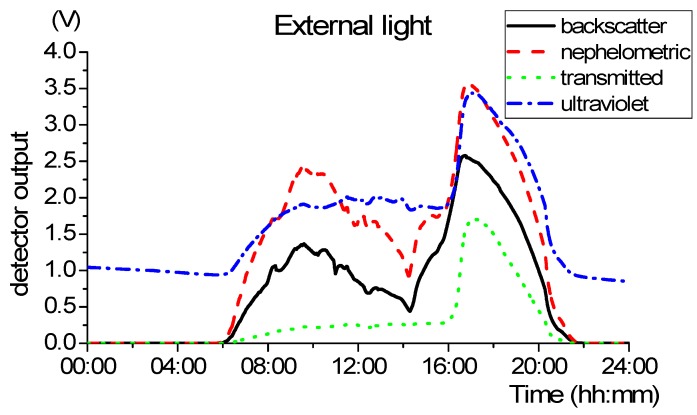
Measurement of the external light (LEDs off) during the first 24 h.

Measurements with the LEDs off were taken to quantify the external light and evaluate their influence on SPM measurements. Figure 12 shows the LEDs off measurements during the first 24 h the sensor was installed, representing the external light influence.

Analysing Figure 12 it is possible to perceive the different periods of the day. During the night, in the period between approximately 21 h and 5 h, the infrared detectors have a null value and the ultraviolet detector is about 1 Volt. These values correspond to the absence of external light. In the remaining periods of the day, from 05:30 the sunrise is observed with the gradual increase of the electrical value of the photodetectors, as well the sunset at 21:00 where the values tend to null. After 16 h, the local where thee sensor is deployed is directly exposed to sunlight.

**Figure 13:**
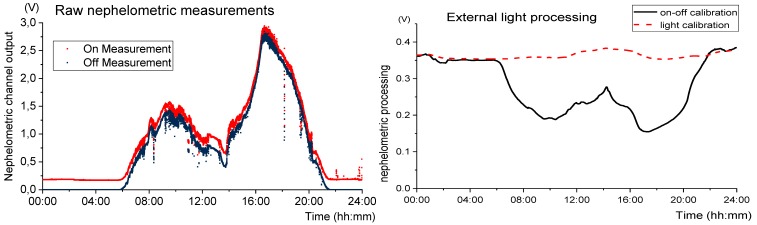
Effect of external light on SPM measurement. In the image on the left, the raw on and off measurements of nephelometric channel (red and blue scatters, respectively) are presented related to the same period of Figure 12. In the image on the right is shown the external light calibration for the on–off technique (solid black line) and for the developed calibration using Equation (14) (dashed red line).

As demonstrated before, this light offset produces erroneous values in turbidity measurements. Figure 13 presents the nephelometric reading before (bottom line) and after (top line) the application of external light calibration, for the same period of Figure 12 (the example of the nephelometric detector is taken, so Equation (14) is used to calibrate the data).

Once again, it is demonstrated that the on–off technique does not eliminate the light effect since the detector output is clearly shaped by the daylight (see Figure 13). In the opposite way, the light calibration presented solves this problem successfully.

### 5.2. SPM Measurements – Tidal Cycles Analysis

As previously demonstrated, the external light has a clear influence on the measurements. Thus, to evaluate SPM monitoring efficiency, the results between 21:30 and 05:30, where there is no external light, are used.

Using Equations (1), (3), (7) and (9), for the correspondent detection technique, results are the following for 180 µm sand calibration.

**Figure 14:**
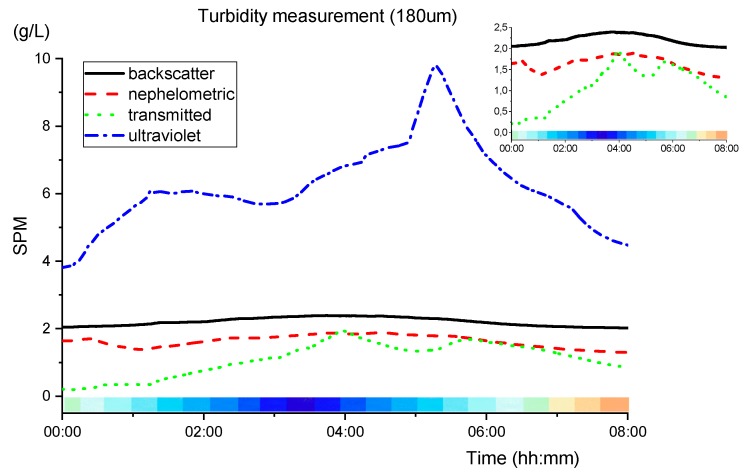
SPM measurements for 180 µm sand, using (1), (3), (7) and (9) calibrations equations, during the night phase (21:30 to 05:30). The colour pattern below the graphs represents the tidal cycle (blue is the low tide at 00:50, and high tide is at 07:00).

Figure 14 shows a slight increase of SPM during the low tide. As expected, the backscatter and nephelometric, due to the principle of operation of measuring the reflected and diffracted light, proved to be the most accurate techniques for low SPM, showing a variation of almost 0.5g/L with the tidal change. In the opposite way, transmitted light has a higher sensitivity due to the direct attenuation of light, which at the minimal obstacle causes a variation in the SPM value.

The ultraviolet transmitted light detection presents a higher SPM value, comparing to the IR detections. That means that organic matter has been sensed, absorbing the UV radiation and creating an offset in turbidity readings. The difference between the IR and UV transmitted light detectors may be used to differentiate inorganic/organic matter. 

**Figure 15:**
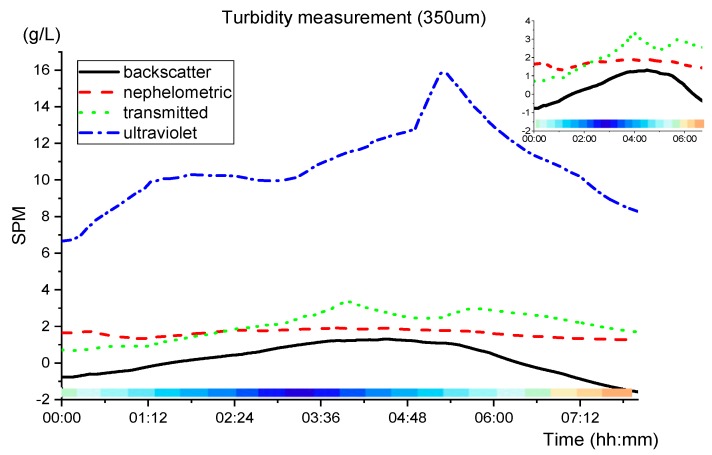
SPM measurements for 350µm sand using (2), (5), (8) and (11) during the night phase (21:30 to 5:30). The colour pattern below the graphs represents the tidal cycle (blue is the low tide at 00:50 and the high tide at 07:00).

For 350 µm sand calibration, using (2), (5), (8) and (11), the results are presented in Figure 15.

Comparing Figure 14 with Figure 15, the nephelometric lines are very similar, however, the backscatter presents negative values and both transmitted detections show higher peaks of turbidity. Not disregarding inaccuracies that may have resulted from the calibration of the sensor, it seems plausible to affirm that the sediments in the local may be closer to 180 µm than to 350 µm.

### 5.3. Biofouling

Biofouling is a problem that any marine/fluvial optical sensor must face [43]. In the immediate moment after the sensor is installed, it is subject to bacterial attachment and biofilm formation on its surface, followed by the attachment of larger marine organisms [44]. For a correct operation of the developed sensor, the emitted light must interact only with the water proprieties. The existence of organic substances or even muds in the optical transducer’s enclosure surface causes an undesired optical attenuation, decreasing its electrical output value [45]. Current state of the art relates to different techniques (coating, active materials, wipers, etc.) used to extend the in-situ monitoring time of optical devices [46,47].

**Figure 16:**
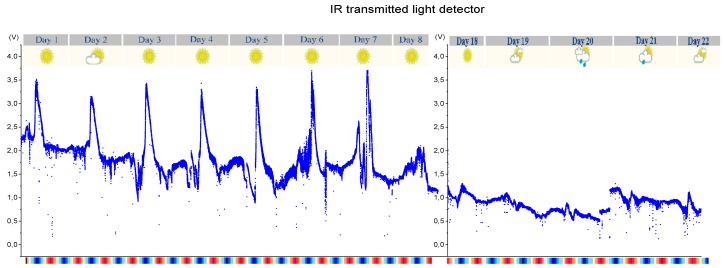
Detector output of transmitted light detector for the first monitoring week on the left and for the last 5 days on the right. The colour pattern below the graph represents the tidal cycle (blue is the low tide and red the high tide).

Figure 16 shows the infrared transmitted light detector’s measures for the first monitoring week (at left) and for the last 5 days (at right). This detector is the most sensitive to light attenuation and therefore the most susceptible to biofouling which leads to a greater variation in SPM measurements over time.

Analysing Figure 16, there appears to be a decrease in the electrical value of the photodetector from day to day. The brightness peaks are not comparable due to atmospheric conditions and sunlight intensity differences for each day; however, analysing the night phase of each day seems to show a gradual decrease of the sensed light. This electrical decrease may not necessarily be a cause of the fouling in the LEDs but an effect of the tides or SPM change; however, analysing all the 22 testing days, it is unequivocal that the optical receiver loses light sensitivity. Comparing the first week data with the last 5 days data, there is a substantial decrease in the electric voltage of the photodetector (about 1 Volt that can represent an error above 20 g/L).

**Figure 17:**
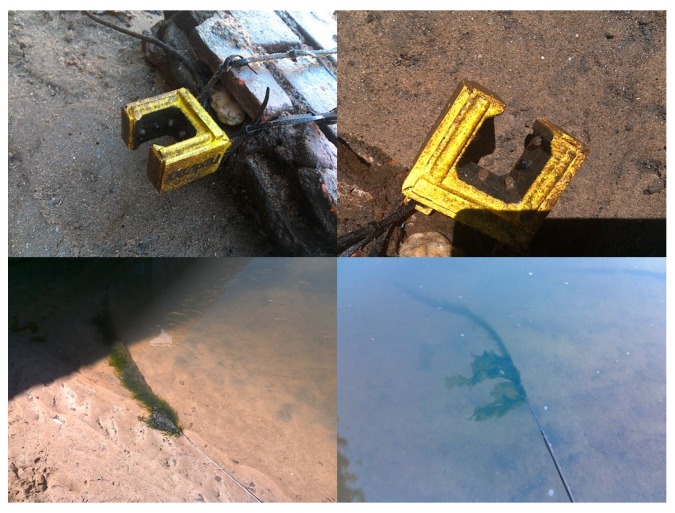
Biofouling in the sensor structure and transmission cable after 22 days of deployment.

After the 22 days of continuous monitoring tests, the sensor was collected and biofouling was visible, not only from biological organisms but also from mud and sand, which were trapped throughout the whole sensor’s structure. The signal transmission cable has also many trapped algae, but no damage in the protective enclosure (Figure 17).

## 6. Conclusions

A low cost and low-power turbidity optical sensor with a production cost in raw materials of less than 20 € was developed for in-situ continuous monitoring. It uses IR backscatter, nephelometric and transmitted light techniques for a wide dynamic range and precision, adapted to different fluvial and marine environments.

In-lab calibrations to suspend particulate matter, organic material (phytoplankton) and formazin turbidity standards were established to prepare the device for in-situ deployments, as well as for external light corrections. SPM readings are strongly dependent on calibration methodology. Seashore sand of 180 µm and 350 µm was used in the SPM calibration to correlate the sensor’s output voltage with SPM. To keep the sediments in suspension, a mechanical mixer was designed, however, some deviation in the measurements could be expected, due to the non-uniform flux and heterogeneity created by the mixer. Further calibration is necessary to improve accuracy. Also, even if the sensor can reach high levels of turbidity (above 4000 NTU), compared to commercial devices available, it still presents a lack of sensibility for low turbidity values. This sensibility can be adjusted, without increasing costs, changing the gain resistors in the hardware design, tuning the nephelometric detector for low turbidity values and backscattering for high turbidity values.

The in-situ monitoring tests showed the potential of the sensor to monitor the concentration of suspended particles, being able to detect suspended particulate matter change with the tidal cycles. The use of an ultraviolet emitter-receptor pair for organic and inorganic matter differentiation has also been shown to be a successful method, provided that an effective calibration is performed. For future work, a deep in-lab calibration must be conducted, with multiple and characterized samples of organic dissolved matter, so that real in-field values of organic load can be estimated. Further in-situ deployment results should also be validated and compared with a ground-truth commercial device.

Biofouling presents the major error associated with optical sensors in a marine environment. Active biofouling protection for optical sensors is under development so that a wide temporal resolution can be achieved without human maintenance.

## Figures and Tables

**Figure A1:**
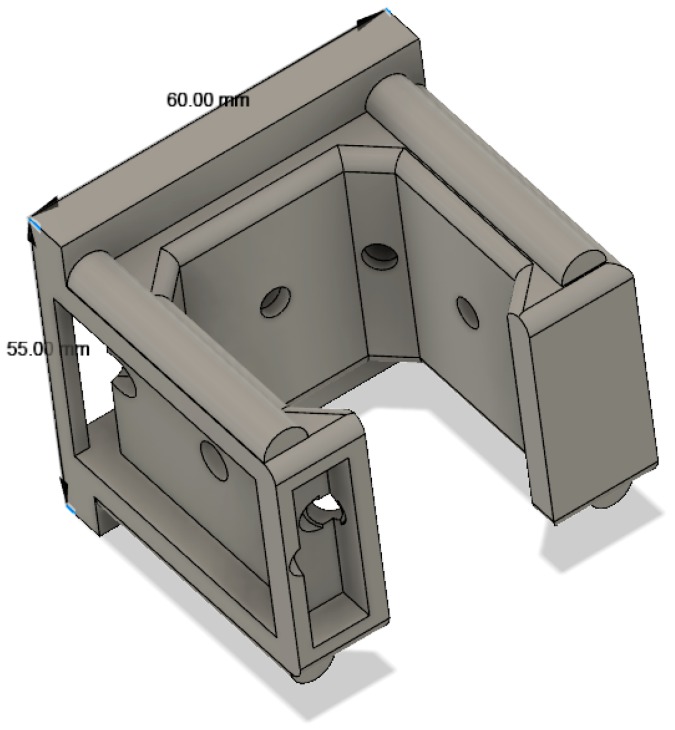
3D model and dimensions of the sensor housing in Fusion360.

**Figure A2:**
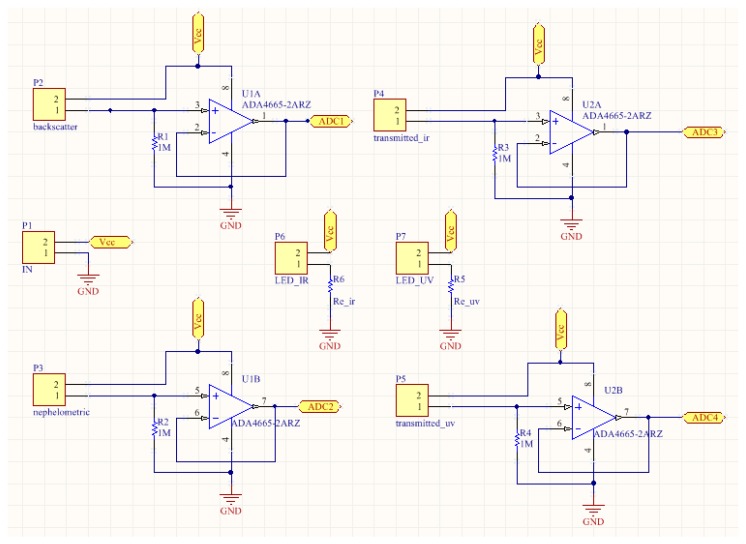
Hardware schematic of the printed circuit board (Altium Designer).

## Appendix A

**Table A1:** Mean and standard deviation results for 180 µm sand calibration. For each concentration, 20 records were made for statistical analysis.

	Backscatter (V)		Nephelometric (V)		Transmitted (V)		Ultraviolet (V)	
g/L	Mean	σ	Mean	σ	Mean	σ	Mean	σ
0	0.186	0.020	0.41	0.012	2.046	0.016	2.075	0.018
10	0.234	0.082	0.527	0.035	1.553	0.215	1.382	0.185
20	0.327	0.023	0.61	0.109	1.235	0.119	0.649	0.153
30	0.352	0.026	0.635	0.046	0.952	0.102	0.356	0.083
40	0.361	0.029	0.664	0.042	0.889	0.092	0.254	0.051
50	0.396	0.093	0.654	0.087	0.659	0.093	0.122	0.033
60	0.425	0.034	0.654	0.101	0.576	0.091	0.015	0.027
70	0.42	0.080	0.635	0.081	0.444	0.081	0	0.012
80	0.469	0.037	0.566	0.053	0.474	0.073	0.01	0.007
90	0.483	0.034	0.527	0.084	0.405	0.084	0.02	0.011
100	0.493	0.056	0.503	0.039	0.381	0.105	0	0.007
110	0.493	0.043	0.464	0.119	0.288	0.046	0.005	0.011
120	0.522	0.090	0.435	0.056	0.264	0.059	0	0
130	0.483	0.057	0.415	0.110	0.225	0.049	0	0
140	0.503	0.039	0.396	0.058	0.19	0.058	0	0

**Table A2:** Mean and standard deviation results for 350 µm sand calibration. For each concentration, 20 records were made for statistical analysis.

	Backscatter (V)		Nephelometric (V)		Transmitted (V)		Ultraviolet (V)	
g/L	Mean	σ	Mean	σ	Mean	σ	Mean	σ
0	0.205	0.019	0.464	0.027	2.144	0.028	2.051	0.022
10	0.229	0.036	0.61	0.072	1.724	0.174	1.675	0.278
20	0.269	0.065	0.698	0.065	1.494	0.200	1.235	0.249
30	0.288	0.072	0.757	0.061	1.265	0.189	0.752	0.236
40	0.366	0.084	0.825	0.078	1.079	0.133	0.547	0.142
50	0.366	0.098	0.791	0.050	0.918	0.060	0.4	0.138
60	0.425	0.043	0.84	0.087	0.718	0.199	0.137	0.180
70	0.41	0.041	0.801	0.056	0.562	0.085	0.107	0.166
80	0.449	0.051	0.854	0.049	0.498	0.101	0.059	0.141
90	0.469	0.099	0.869	0.083	0.396	0.204	0.039	0.049
100	0.508	0.093	0.894	0.066	0.293	0.058	0.015	0.079
110	0.498	0.088	0.903	0.052	0.273	0.045	0.029	0.026
120	0.444	0.044	0.84	0.087	0.237	0.055	0	0.014
130	0.415	0.039	0.859	0.071	0.225	0.052	0	0.007
140	0.444	0.051	0.762	0.052	0.215	0.035	0	0.007
